# Actively cycling cells in uninjured connective tissue are not a prerequisite for appendage regeneration

**DOI:** 10.64898/2026.07.25.740716

**Published:** 2026-07-27

**Authors:** Emilio A. Oviedo-Rivadeneira, Ashley W. Seifert

## Abstract

Multiple hypotheses have been formulated to explain differences in tissue repair ability across vertebrates. One hypothesis posits that the accessibility of actively cycling stromal cells within uninjured tissue confers access to a proliferative population in response to tissue damage. This hypothesis further suggests that animals with an indeterminate growth mode possess an actively cycling cell population necessary for growth that can be readily accessed for tissue regeneration. Moreover, the absence of an actively cycling population in connective tissue provides a mechanism that restricts regeneration in animals with determinate growth whose cells are refractory to cell cycle progression and proliferation to produce new tissue for morphogenesis. Here, we explore this paradigm using an EdU-BrdU pulse chase strategy in four different vertebrate species: two with determinate ( *Acomys dimidiatus* and *Mus musculus* ) and two with indeterminate modes of growth ( *Danio rerio* and *Ambystoma mexicanum* ). We find that although indeterminate growers do possess a small population of actively cycling cells, this population does not contribute to regeneration. Moreover, we found that while *Acomys* does not possess a population of actively cycling stromal cells, cells re-enter the cell cycle *de novo* in these animals to contribute to regeneration. Furthermore, testing this hypothesis allowed us to ask whether tissue injury could stimulate cell cycle re-entry – a so-called *primed* state - in cells at distance from the injury site in these four species and we did not find evidence of such priming in stromal or epidermal tissue.

**Highlights:**

- Cell cycle re-entry is a common response to injury in regenerative and non-regenerative vertebrates that is independent of actively cycling stromal cells in uninjured connective tissue
- Actively cycling cells do not contribute to regenerative healing in spiny mice, axolotls or zebrafish.
- Our data do not support systemic cell cycle activation in response to injury.

## Full Text Availability

The license terms selected by the author(s) for this preprint version do not permit archiving in PMC. The full text is available from the preprint server.

